# Gut microbiota of red swamp crayfish *Procambarus clarkii* in integrated crayfish-rice cultivation model

**DOI:** 10.1186/s13568-019-0944-9

**Published:** 2020-01-14

**Authors:** Yan Shui, Zheng-Bing Guan, Guo-Feng Liu, Li-Min Fan

**Affiliations:** 1 Key Laboratory of Freshwater Fisheries and Germplasm Resources Utilization, Ministry of Agriculture, Freshwater Fisheries Research Center, Chinese Academy of Fishery Sciences, 9# Shanshui East Road, Wuxi, 214081 Jiangsu China; 2 The Key Laboratory of Industrial Biotechnology, Ministry of Education, School of Biotechnology, Jiangnan University, Wuxi, 214122 Jiangsu China; 3 Wuxi Fisheries College, Nanjing Agricultural University, Nanjing, China

**Keywords:** Integrated crayfish-rice cultivation model, Gut microbiota, *Procambarus clarkii*, Illumina MiSeq sequencing

## Abstract

Increasing evidences suggest that intestinal microbiota balance closely correlated with host’s health status could affected by external environment. Integrated crayfish-rice cultivation model is a highly efficient artificial ecosystem widely practiced in subtropical China. Less information is available to estimate the influence response to the micro-ecology of crayfish intestine and so as to influence the biological processes. Thus, 16S rRNA high-throughput sequencing approach was employed to investigate the composition diversity and functions of bacterial community in the intestines of *Procambarus clarkii* farmed within this model. Results exhibited the highly diversity of microflora with dominant phyla *Actinobacteria*, *Proteobacteria*, *Tenericutes*, *Firmicutes* and *Bacteroidetes*. The genera of *Candidatus Bacilloplasma* and *Ornithinibacter* were presented as predominant population much exceeds in richness comparing to that of other genus. Despite the highly diversity in the bacterial community, the predicted functions indicated relative consistent in biological processing pathway. Collectively, significant richness of genes was observed involved in amino acid and carbohydrate metabolism and membrane transport processing. This study would contribute to the understanding of the impact of growth conditions on host–microbiota relation especially in aquatic animals.

## Introduction

The red swamp crayfish *Procambarus clarkii* is a highly adaptable and fecund freshwater crayfish species becoming an important aquatic food sector among fishery trading commodities in China (Du et al. 2017; Shen et al. 2014). The production exceeded 1 million tons with 37 billion USD output in 2017, accounting for 80% of global production according to FAO statistics (FAOSTAT, http://www.fao.org/faostat/en/) and Crayfish Industry Report 2018 (http://www.moa.gov.cn/). This is in contrast to the reality that it considered as an invasive species in Europe and Asia (Carreira et al. 2017). The vigorous development of the industry was substantially attributed to the improved optimization of efforts involved in crayfish-rice farming, which original model has been practiced in Louisiana, USA for several decades (Si et al. 2017a; Anastacio et al. 1999).

In this integrated crayfish-rice cultivation (CR) model, wastes (e.g. weeds, insects, leafs, and plankton) utilized as food sources be eaten by crayfish. While crayfish dig burrows increasing soil permeability and material/energy circulation and its feces used as high-quality fertilizer and thereby, advance the rice growth. Literatures have demonstrated that CR model significantly affects chemical and biological processes of belowground and increase soil microbial biomass comparing to the monoculture (Si et al. 2017b; Cai et al. 2019). In 2018, CR model has been practiced to an area of approximately 200,000 hm^2^ mostly in subtropical China, showing the significant economic and social benefits (Cao et al. 2017). Modeling of CR ecosystem has been a widely explored topic, and many papers have been presented (Si et al. 2017a; Barbee et al. 2010; Li et al. 2018a). In general, these efforts have primarily focus on the establishment to the effect of environment utilization and energy/materials benefits.

Increasing evidences pointed to a strong association between colonized microorganisms and biological processes (e.g. metabolism, immunity response, et al.) of host especially in aquatic species (Gómez and Balcázar 2008; Roeselers et al. 2011). In *Litopenaeus vannamei*, significant difference in the microbial composition and function was observed after WSSV infection, suggesting the intestinal microbiota function in immune response (Wang et al. 2019). Studies in Rainbow trout indicated that insect meal positively modifies gut microbiota, increasing its richness and diversity and in particular, increasing the amount of beneficial lactic acid and butyrate producing bacteria (Terova et al. 2019). Moreover, Li et al. (2018b) pointed out that the gut microbiota of Asian carp depends on exact species, even when cohabiting in the same environment. These findings suggesting the importance but complexity functions of gut microbiota in the aquatic animals affected by various external interferences. CR model is indeed a highly efficient artificial ecosystem organic combining agriculture and aquaculture together. From the microbial point of view, investigating the diversity and functions of microbiota in vivo of the crayfish habiting in such environment, would contribute to the understanding of the impact of ecology on crustaceans and perhaps, to improve culture strategies. To date, less information is available however.

Accordingly, in the present study, we employ the Illumina MiSeq high-throughput sequencing (HTS) technologies and assembly analysis targeting the V3–V4 region of the bacterial 16S rRNA gene to investigate the composition, diversity and function dynamics of *P. clarkii* gut microbiota involved in the growth condition caused by the CR model, as well as providing a theoretical basis for promoting the system. To our knowledge, this study is the first regarding to the gut bacterial community of crayfish farming within CR model via HTS.

## Materials and methods

### Study site and sample collection

This study was conducted on the up-to-10 years practiced CR model in waterlogged rice paddy fields in Huai’an City, Jiangsu Province, China. The region has a humid, subtropical monsoon climate with a 1500 mm average annual rainfall. A peripheral trench (3.0 × 1.5 m) was excavated and crayfish larvae (weighing 5 ± 2 g) were stocked at a density of 1.5 × 10^5^ larvae·ha^−1^, and the crayfish self-propagated inside the rice paddies.

Healthy and robust crayfish weighing approximately 15–25 g were collected from this ecosystem. They were acclimatized at ambient temperature (22 ± 1 °C) in air-pumped circulated artificial freshwater before the treatment. Five individuals were randomly captured and the intestine was sampled respectively (mark with S1–S5). The intestine was separated and its contents were collected using sterilized equipment, in which there was no contact with soil or other pollution sources. All samples were stored at − 80 °C immediately before DNA extraction.

### DNA isolation, PCR amplification, and Miseq sequencing

Total genomic DNA was isolated from intestine tract samples using the Qiagen QIAamp Fast DNA Stool Mini Kit according to the instructions. The concentration of isolated DNA was measured by a Nano-Drop 2000 spectrophotometer (GE Healthcare, USA). PCR amplification of the 16S rRNA gene was performed based on the literature (Liu et al. 2008) using PCR primers specific for the V3–V4 regions. All PCR products were visualized on agarose gels (2% in TAE buffer) containing ethidium bromide, and purified with a DNA gel extraction kit (Axygen, China). Prior to sequencing, the DNA concentration of each PCR product was measured by spectrophotometer and mixed with the appropriate proportion based on sequencing requirements. Amplicon libraries were constructed, and sequencing was performed using the Illumina Miseq platform at Majorbio Bio-Pharm Technology Co., Ltd., Shanghai, China.

### Sequence analysis

Sequences from raw data were analyzed and filtered by Quantitative Insights into Microbial Ecology as reported previously (QIIME, http://qiime.org/index.html). Sequencing error and chimeras were detected and removed; reads that could not be assembled were discarded. Sets of sequences with at least 97% identified were defined as an operational taxonomic unit (OTU) by Uparse (http://drive5.com/uparse/). The highest frequency sequences in OTUs act as the representative sequence. GreenGene Database (http://greengenes.lbl.gov/) was employed to align the sequences. The taxonomic information was annotated by RDP classifier (http://sourceforge.net/projects/rdp-classifier/) with 70% confidence threshold (Quast et al. 2013). Data of OTUs abundance was normalized by the standard sample with the least sequences. Subsequent analysis of alpha diversity and beta diversity were all performed basing on the normalized data.

### Statistical methods

Rarefaction curves were plotted for each sample to determine the abundance of communities and sequencing data of each sample. Alpha-diversity analyses, including community richness parameters (Chao1, ACE, Rank-Abundance curves), community diversity parameters (Shannon, Simpson, Shannon–Wiener curves), and a sequencing depth index (Good’s coverage), were calculated using the Mothur software (Yang et al. 2014). Beta diversity measurements including Approximately Maximum Likelihood phylogenetic trees mapped using FastTree (http://www.microbesonline.org/fasttree/) as well as principal coordinate analyses (PCoA) based on OTU compositions were calculated. Unweighted pair-group method with arithmetic means (UPGMA) tree was contrasted via QIIME. A Venn diagram conducted by Draw Venn Diagram online (http://bioinformatics.psb.ugent.be/webtools/Venn/) was implemented to show unique and shared OTUs. Differences between populations were analyzed using a one-way ANOVA; *p* < 0.05 was considered statistically significant.

OTU tables were generated by closed-reference picking protocol to predict the metagenomes using Phylogenetic Investigation of Communities by Reconstruction of Unobserved States (PICRUSt). After normalized by the 16S rRNA copy numbers, functional pathways were predicted by Cluster of Orthologous Groups (COG) of proteins annotation as well as Kyoto Encyclopedia of Genes and Genomes (KEGG) catalogue at level 1, 2 and 3 KEGG orthology groups (KOs).

### Accession number

Raw data of all samples in this study has been deposited in Sequence Read Archive database of NCBI under the SRA Accession: PRJNA557576.

## Results

### Sequencing depth and alpha diversities

Totally, we obtained 279,880 high-quality sequences from the intestine content of 5 crayfish samples within CR model. The sequencing reads range from 52,552 to 60,178, with an average of 55,976 reads per sample. For the further downstream analyses, sequencing reads was normalized to minimum (43,880 reads) by randomly subsampling to standardize sampling effort. These normalized sequences with average length of 418 bp were assigned to 646 OTUs (163–470) with 97% sequence similarity (Table 1 and Additional file 1: File S1). Good’s coverage regarded as the indicator to estimate the completeness of sequencing was calculated among the samples, ranging from 99.75 to 99.80%. The rarefaction curve at an OTU definition of 97% identity and Rank-Abundance curves were shown in Additional file 2: Figures S1 and S2 respectively.

**Table 1:** Number of reads, statistical estimated community richness index (chao and ace), community diversity index (simpson and shannon), and Good’s coverage for 16S rRNA libraries of *P. clarkii* intestinal microbial ecosystems

Sample No.	Number of seqs	Read number	OTUs	Chao	Ace	Simpson	Shannon	Good’s coverage
S1	52,552	43,880	272	365.53	370.52	0.34	1.85	99.80
S2	54,533	43,880	163	230.20	326.26	0.16	2.26	99.85
S3	56,711	43,880	470	556.67	568.28	0.06	3.67	99.76
S4	60,178	43,880	439	559.38	555.35	0.04	3.96	99.75
S5	55,906	43,880	363	446.07	443.70	0.09	3.16	99.80

To investigate the difference of community richness and diversity, alpha diversity index including chao1 and ace (community richness indexes), Simpson and Shannon (diversity indexes) were also calculated. The indexes of diversity at a genetic distance of 3% are presented in Table 1. No significant difference in alpha diversity indices were observed among the samples (*p* > 0.05).

### Taxonomic composition

**Fig. 1:**
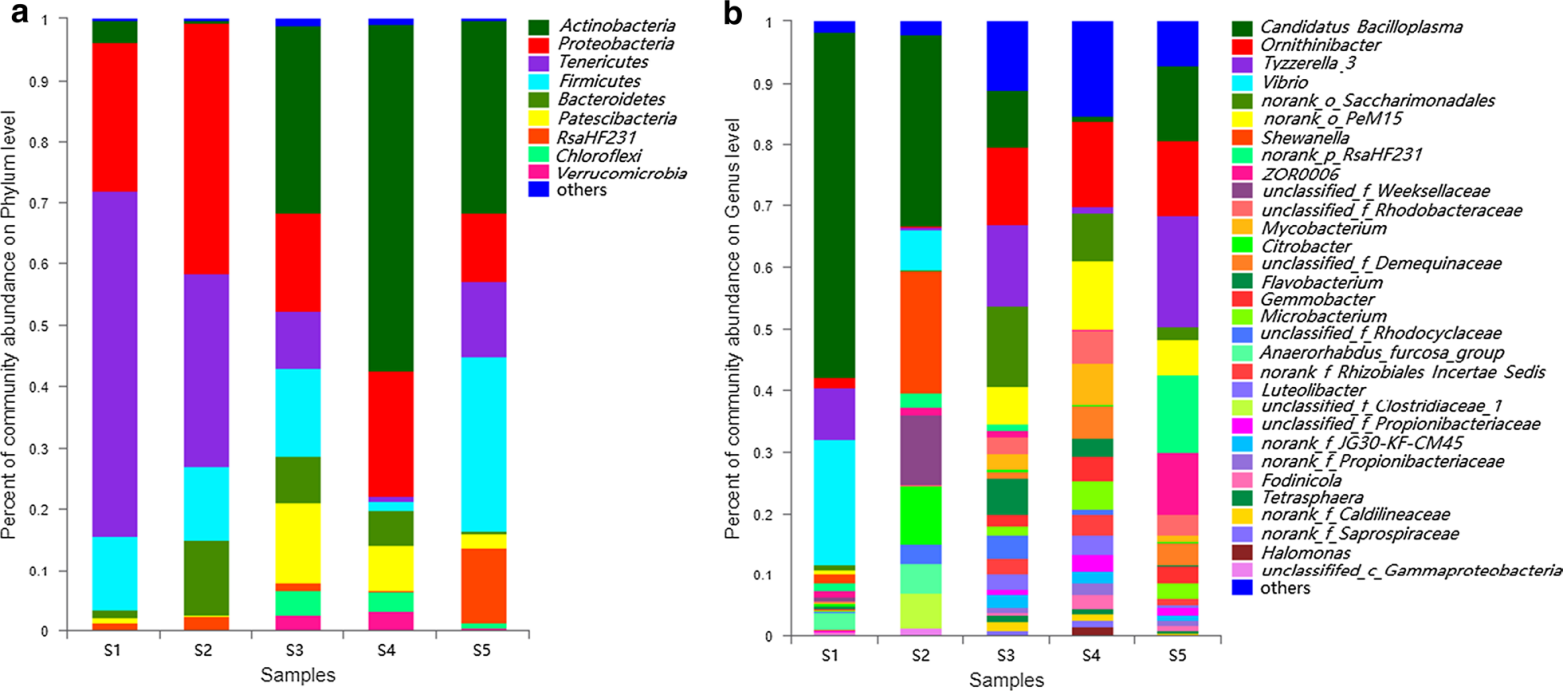
Community barplot analysis showing the relative abundance of gut bacterial community among the samples by Mothur at the phylum (**a)** level and genus (**b)** level. Less than 1% abundance of the phyla/genera was merged into “others”

A total of 19 taxa were identified at the phylum level in this study (Fig. 1a). Significant differences were observed in the relative abundance of gut microbiota among the samples (*p* < 0.05). Top five phyla in abundance were considered to be the core gut microbiota: *Actinobacteria* (range from 0.41–56.47% in each sample); *Proteobacteria* (11.21–41.07%); *Tenericutes* (0.94–56.13%); *Firmicutes* (1.40–28.30%); and *Bacteroidetes* (0.51–12.40%). In general, these core microbiotas accounted for roughly 80% of the total in the samples S3, S4 and S5; but 97% in other samples (S1 and S2). There was some variation in microbial composition in more detail. For instance, microbiota from sample S2 demonstrated a markedly lower abundance of *Actinobacteria* (0.41%) in its intestine than that of others (3.74, 30.83, 31.72, and 56.47% respectively); while microbiota from sample S4 showing a much lower abundance of *Tenericutes* (0.94%) than that of others (9.36, 12.22, 31.31, and 56.13% respectively).

As shown in Fig. 1b, a large number of sequences were classified (98.30, 97.88, 88.92, 84.78, and 92.87% in samples S1–S5, respectively), and more relative differences were presented at the genus level. Altogether, detected OTUs were distributed among 267 bacterial genera. In the composition of sample S1, *Candidatus Bacilloplasma* was distinctly the predominant (56.12%), followed by *Vibrio* (20.31%) and *Tyzzerella 3* (8.30%). Actually it was also the main component genus in sample S2 (31.31%); others are *Shewanella* (19.67%), *Unclassified Weeksellaceae* (11.38%), *Citrobacter* (9.40%), et al. The abundance of *Tyzzerella 3* in sample S3 and S5 was both around 16.95%, but other samples (S1, S2 and S4) had hardly any of this genus. *ZOR0006* was present in sample S5 with the abundance of 10.08%, while averagely lower than 0.5% in other samples. Interestingly, we found that *Unclassified Weeksellaceae* was common to sample S1 and S2 but not present in others.

### Community structures

**Fig. 2:**
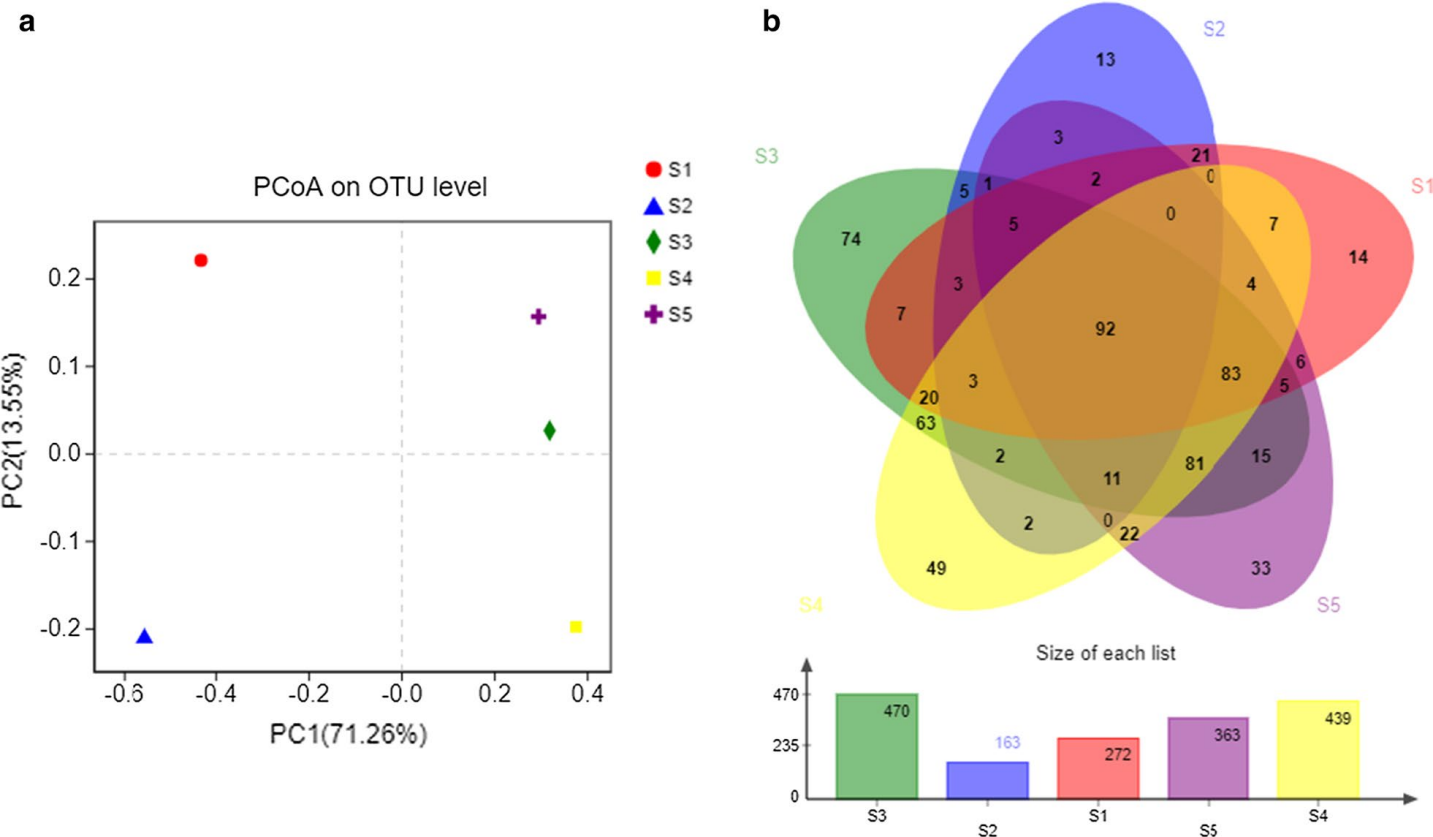
Principal coordinates analysis (PCoA) and the Venn diagrams. **a** PCoA of gut microbial communities on OTU level, each symbol represents one gut microbiota, and distance between symbols on the ordination plot reflect relative dissimilarities in community structures; **b** the shared and unique OTUs were represented through Venn diagrams. Venn diagram at distance 0.03; 92 OTUs were shared among samples

We explored the PCoA analysis to examine the community structures of the gut microflora among the samples at the OTU level (Fig. 2a). On the PCoA plot, each symbol represents one gut microbiota, and distance between symbols on the ordination plot reflects relative dissimilarities in community structures. The result demonstrated that microflora communities of sample S3, S4 and S5 clustered tightly and, were separated from those of sample S2 and S1 along principal coordinate axis 1 (PC1), which explained the largest amount of variation (71.26%). This result is also agreement with the taxonomic composition analysis, where samples S1 and S2 were found to possess a significantly lower number of OTUs than those of samples S3, S4 and S5.

To further compare the diversity and composition of gut microbiota, the shared and unique OTUs were represented through a Venn diagrams (Fig. 2b). Pairwise comparison was performed among the samples by considering a similar level of 97% OTUs for analysis. As shown in the figure, across samples, they exhibited relatively similar with 92 OTUs overlapping.

**Fig. 3:**
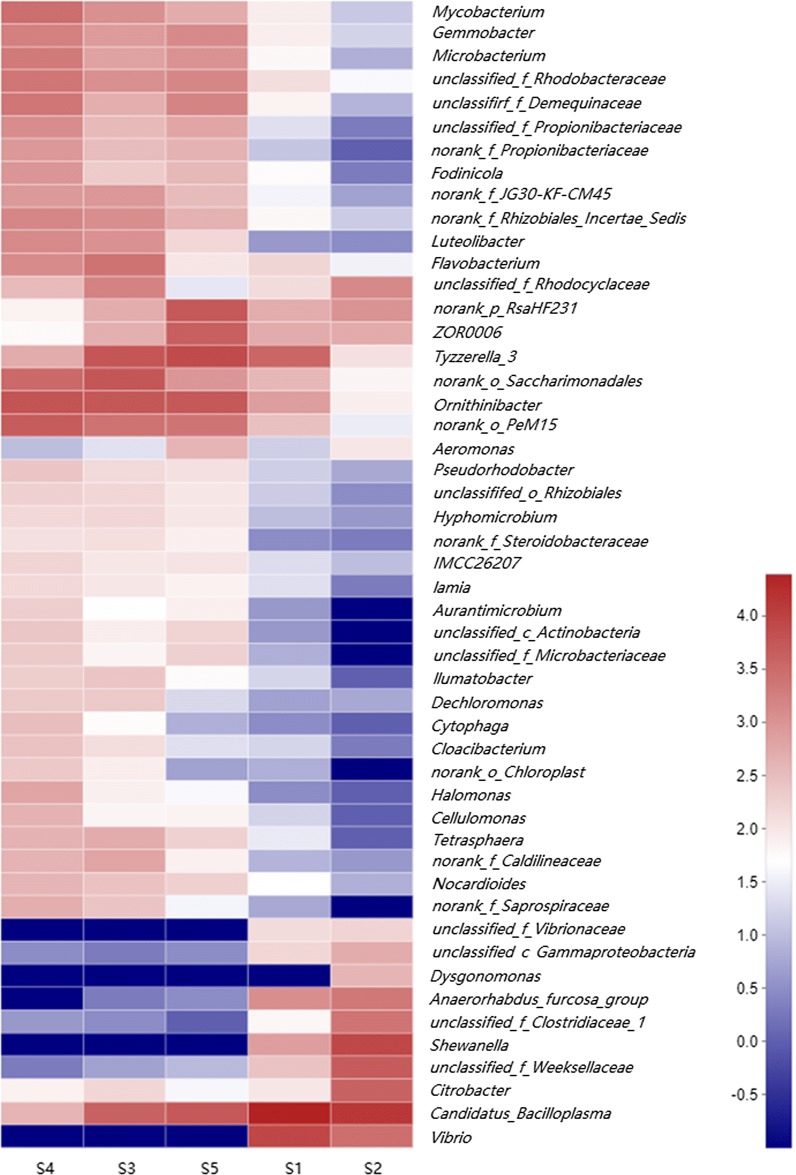
Heatmap to estimate the similarities of the membership and structure of the samples at the genus level. The color code represented the similarly of the sample gut microbe communities, where blue (value =−1) represents the lowest and the red (value = 4.5) the highest level of abundance

In addition, a community Heatmap was plotted to estimate the similarities of the membership and structure among the samples at the genus level (Fig. 3). On this map, red indicates communities that are more similar than those colored in blue. The result reveals that the highest degree of similarity of the taxonomic composition was presented among samples S3, S4 and S5, which distinguishes from the rest two samples (composition of S1 and S2). Correspondingly, the additional Hierarchical clustering tree at the OTU level was presented showing the consistent result with the Heatmap result above (Additional file 2: Figure S3).

### Evolutionary analysis

**Fig. 4:**
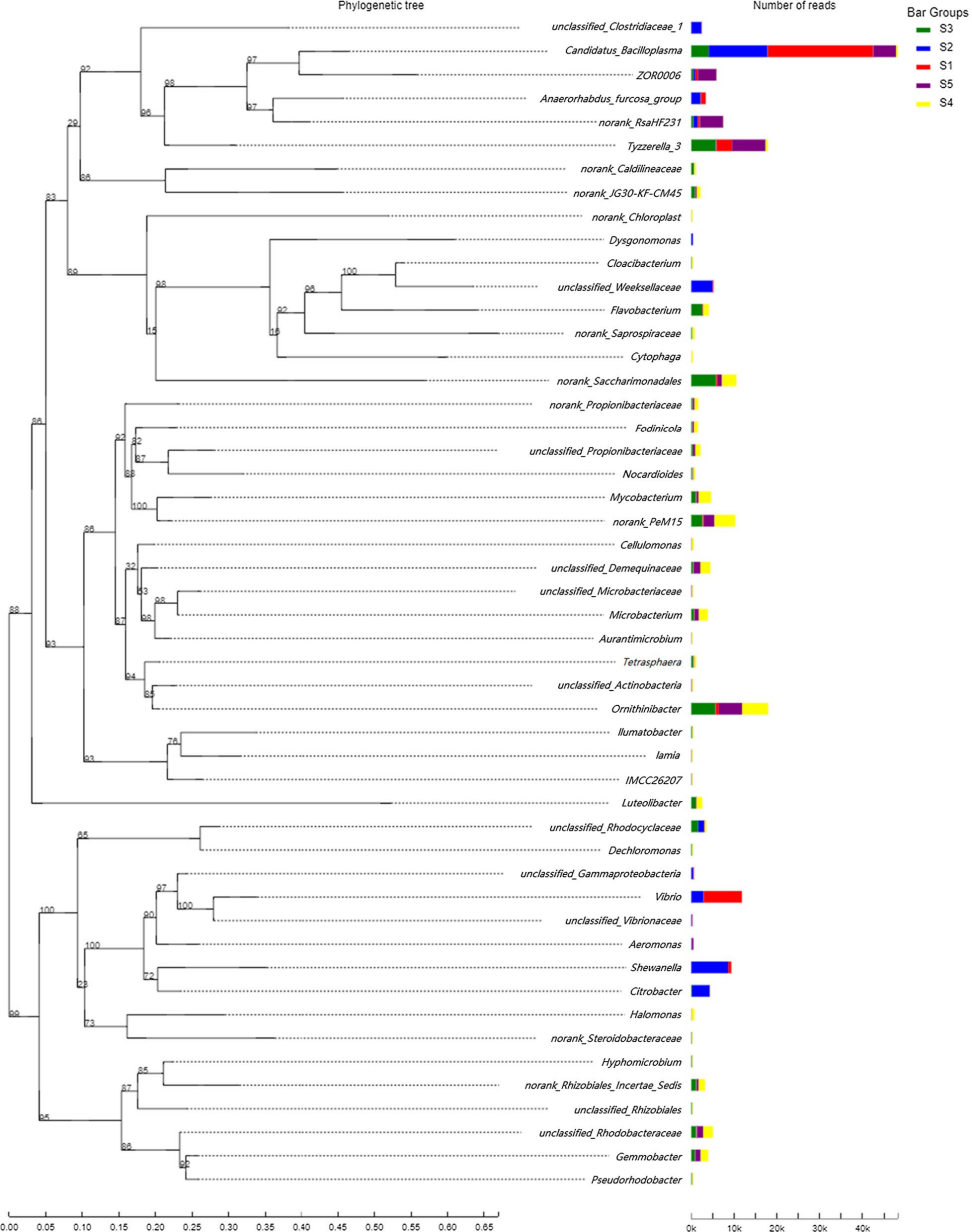
Phylogenetic tree showing the phylogenetic relationship among the samples on genus bar. All bootstrap values > 50% was shown on the tree

On the basis of sequence similarities, we employed the FastTree software to map the phylogenetic trees at the genus (Fig. 4) and phylum level (Additional file 2: Figure S4). The results were presented in the form of a combination of evolutionary trees as well as reading abundance. As detailed in these figures, the sequences were classified into several clusters corresponding to the major genus and phylum, the domain bacteria. *Tenericutes*, *Firmicutes*, *Actinobacteria* and *Proteobacteria* represented the top reading abundance, which is consistent with the above taxonomic composition analysis (Fig. 1). The phylum *Proteobacteria* uniform distributed among samples showing a relatively distant genetic distance. Across the samples, *Candidatus Bacilloplasma* in phylum *Tenericutes* was presented as predominant population much more in richness comparing to that of other genus.

### 16S rRNA functional prediction analysis

To analyze the functions of gut microbiota among the samples, metagenomes potential were predicted by PICRUSt. Sequences were conducted and submitted to the KEGG database for analysis. Results revealed the high abundance of bacterial metagenomes in crayfish intestine (Additional file 3: File S2). Briefly, they were mainly associated with Metabolism pathway group, Genetic information processing group, Environmental information processing group and the so-called unclassified group in KEGG level 1 (Table 2). For the Metabolism group, the largest abundance was found in Amino Acid Metabolism, Carbohydrate metabolism, Energy Metabolism and Metabolism of cofactors and vitamins et al. in level 2. For the Genetic Information Processing group, abundance was found in Replication and Repair, Translation, as well as Transcription. Significantly abundance was observed in Membrane Transport pathway group belonging to the Environmental Information Processing group in KEGG level 1.

**Table 2:** Abundance of predicted functions in KEGG database

Level	Pathway pattern	S1	S2	S3	S4	S5
1	Metabolism	51.98	44.96	53.29	53.84	52.75
2	Amino acid metabolism	10.90	8.94	11.45	11.65	21.06
2	Carbohydrate metabolism	10.87	8.85	10.95	11.28	11.19
2	Energy metabolism	5.72	5.35	5.79	5.60	5.68
2	Metabolism of cofactors and vitamins	4.13	4.23	4.27	4.26	4.21
2	Xenobiotics biodegradation and metabolism	3.92	2.36	4.23	4.46	3.99
2	Lipid metabolism	4.04	3.16	4.21	4.22	4.03
1	Genetic information processing	17.13	17.59	15.63	15.50	16.99
2	Replication and repair	7.91	7.73	7.14	7.10	7.90
2	Translation	4.70	4.79	4.05	3.96	4.60
2	Transcription	2.32	2.49	2.29	2.34	2.34
1	Environmental information processing	14.42	14.22	14.59	14.76	14.87
2	Membrane transport	12.32	11.11	12.46	12.78	12.97
2	Signal transduction	1.89	2.96	1.93	1.80	1.70
1	Unclassified	12.16	16.44	12.09	11.72	11.39
1	Cellular processes	2.45	4.68	2.49	2.24	2.13
1	Human diseases	0.87	1.26	0.87	0.85	0.84
1	Organismal systems	0.80	0.62	0.85	0.89	0.84

Parts of KOs in KEGG level 1 and level 2 were listed

**Fig. 5:**
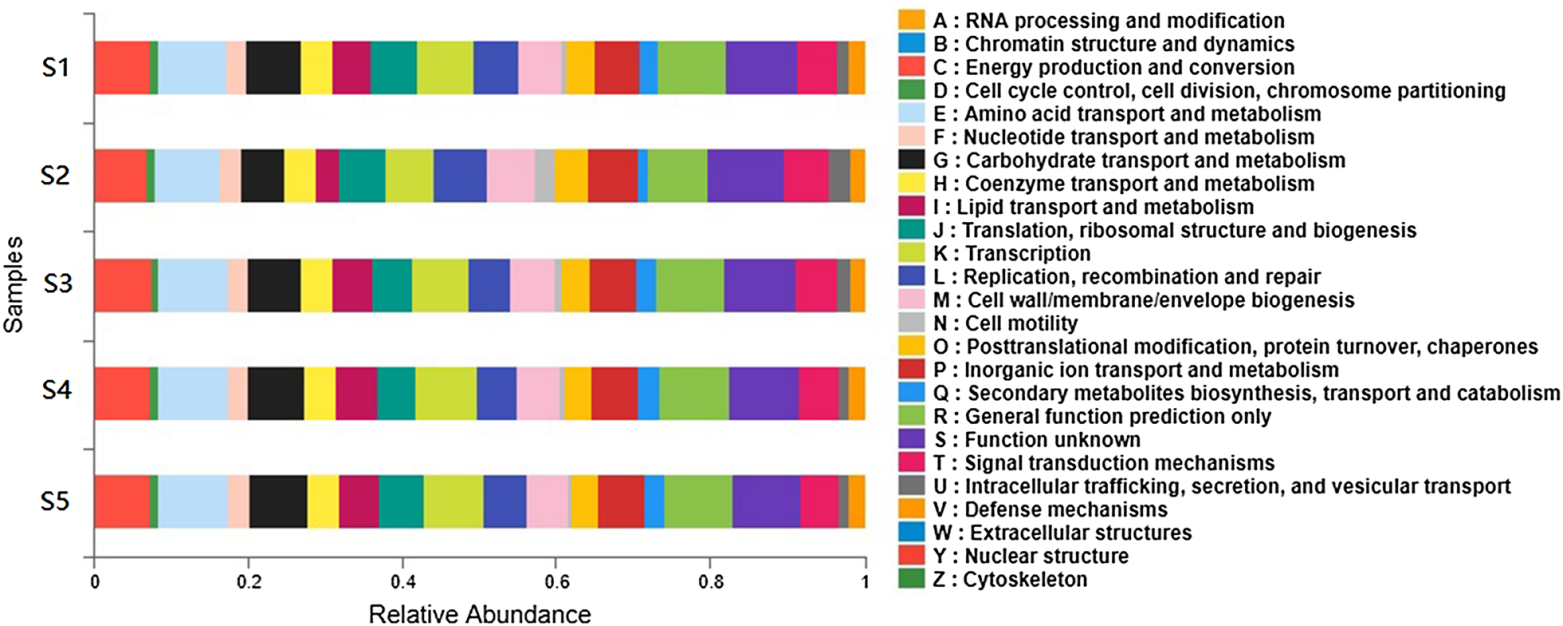
Relative abundance of COG function classification for each samples were predicted by PICRUSt

Sequence similarity search was also conducted and all clusters were submitted to the search against the cluster of COG database for functional prediction and classification in this study. Among the 25 COG categories shown in Fig. 5 and Additional file 2: Figure S5, the relative abundance were observed in “function unknown” group (9.13, 9.96, 9.32, 9.06 and 8.84% in sample S1–S5 respectively), “general function prediction only” group (8.83, 7.72, 8.82, 8.92 and 8.90%), “amino acid transport and metabolism” group (8.81, 8.44, 9.05, 9.02 and 9.03%) and “Energy production and conversion” group (7.13, 6.73, 7.35, 7.28 and 7.20%), with the following “extracellular structures” and “nuclear structure” groups being the smallest abundance.

## Discussion

As has been generally accepted, the bacterial diversity is closely associated with the functional stability of endogenous microbiota in aquatic fauna (Pan et al. 2019). Many factors (age, genotype, diets, and habitat, etc.) affect the community architecture; thereby the dominant bacteria are well recognized compete to maintain a well-balanced intestinal flora continually (Cresci and Bawden 2015). Additionally, understanding the principles governing microbial community assembly and its maintenance within the intestine had led to a reflection measuring the ecological significance of the ecosystem. Currently, cultivation of *P. clarkii* within CR model presents great potential both economically and ecologically. In this work, attention was paid to the composition diversity and the function of gut microbiota in *P. clarkii* within CR model. Using the 16S rRNA Illumina MiSeq sequencing approach, a total of 19 phylotypes as well as 267 genera were observed, showing the highly diversity and richness of intestinal microflora.

In general, *Actinobacteria*, *Proteobacteria*, *Tenericutes*, *Firmicutes* and *Bacteroidetes* constituted the predominant phyla in this work, which corroborated with the previous studies on crustacean species (Hoyoux et al. 2009; Abdelrhman et al. 2016; Egerton et al. 2018). These evidences have indicated that *Actinobacteria* is normally the most abundant phyla in *P. clarkii* intestine. Functionally, *Actinobacteria* was regard to participate in the decomposition of organic matter in soil and sediments, and studies in mammals have shown a positive correlation with fiber intake (Henning et al. 2017). While some carp species (Li et al. 2018b), birds (Sun et al. 2018) and mammals (Gu et al. 2013; Andersson et al. 2008) shared *Bacteroidetes* as the most common phylum (relative abundance > 10%), *Firmicutes* (mostly the genus of *Tyzzerella 3* in this work) was found with relative abundance evenly distributed among the detected samples. Of relevance, literatures have indicated that the *Firmicutes* (*Proteobacteria* as well) might be urgent for some physiological and biochemical functions of the intestine of crustacean species (Zeng et al. 2016). Furthermore, the phylum *Firmicutes* which potential metabolizes dietary plant polysaccharide in the mammal gut was regarded as the most abundant phylum in the feces microflora of carnivorous animals (Becker et al. 2014; Wu et al. 2016). This could explain at least in part its existence in *P. clarkii* intestine samples in this work, as crayfish is a carnivorous freshwater species with extensive feeding patterns. In short, *P. clarkii* cultivation within CR model provided extra in rich source of food (e.g. weeds and insects) for the species in comparing with the monoculture feeding with commercial feed (Si et al. 2017a).

In terms of the genus, *Candidatus Bacilloplasma* was found as the dominant with significant high abundance in this work, followed by *Ornithinibacter*, *Tyzzerella 3*, *Vibrio*, *Norank Saccharimonadales* et al. These findings were partly consistent with the previous notable research on crayfish intestinal, which also listed the major genera (*Candidatus Bacilloplasma*, *Bacteroides*, *Vibrio*, *Acinetobacter*, *Dysgonomonas*, *Tyzzerella 3*, *Aeromonas* et al.) (Feng et al. 2019). *Candidatus Bacilloplasma* was considered to be the novel lineage of *Mollicutes* associated with the hindgut wall of the isopod (Crustacea: Isopoda) first identified in *Porcellio scaber* (Kostanjsek et al. 2007). Literatures also demonstrated that *Candidatus Bacilloplasma* OTUs were clustered into several subgroups in which the neighboring sequences were obtained from a variety of sea animals but mostly in crustacean [*Litopenaeus vannamei* (Rungrassamee et al. 2016), *Eriocheir sinensis* (Chen et al. 2015), *Pelteobagrus fulvidraco* (Wu et al. 2012) and *Nephrops norvegicus* (Meziti et al. 2010)]. These findings suggested that this population was highly diversified and played significant roles in the gut microbiota of crustaceans. Nevertheless, no isolated strain is available yet. Of note is the significant abundance of *Ornithinibacter*, which occupying to the dominant of phyla *Actinobacteria*. To the best of our knowledge, no evidence with such high abundance of this population was found in other studies. Given that all intestinal samples in this work were collected from healthy adult species, the potential role of *Ornithinibacter* in these samples was unclear but could be associated with the pathway of genetic information processing. Meanwhile, it is also unknown whether the high abundance of *Candidatus Bacilloplasma* and *Ornithinibacter* observed in this work is associated with the growth condition caused by CR model cultivations. The effect of these bacteria in *P. clarkii* intestine needs further investigation.

At the genus level we identified several extra pathogens in the healthy *P. clarkii* intestine, such as *Vibrio*, *Aeromonas* and *Bacteroides*. These pathogens recognized as the Gram- facultative/oblige anaerobic bacteria which were commonly existed in the variety of marine and freshwater species (Egerton et al. 2018; Di et al. 2014), suggesting the endogenous microbial as members of the established-flora associated with aquatic environment corresponding to the transient-flora. Actually, studies in *L. vannamei* have indicated that bacterial diseases due to *Vibrio* species in shrimps are often associated with grow out conditions (Wang et al. 2019). The dominant position of these pathogens in healthy *P. clarkii* intestine indicated that pathogenic bacteria may act as opportunistic agents in causing diseases. No member of *Cyanobacteria/Chloroplast* or Lactic acid bacteria was detected in *P. clarkii* intestinal samples in this work, suggesting that *Cyanobacteria/Chloroplast* indeed not to be a food source for crayfish although it is in most fish species (Dong et al. 2014).

In line with other studies on aquatic hosts such as *Danio rerio* (Burns et al. 2016), *Scylla serrata* (Augusto and Serrano 2012) and *Eriocheir sinensis* (Sui et al. 2015), results from UPGMA clustering, Heatmap and PCoA for the community structure consistently demonstrated that *P. clarkii* under identical conditions did not possess the same microflora in similar proportions and particular, in the most abundant phyla. These findings suggested a general phenomenon in adult animals. Given that the individuals were reared under the same conditions, the differences in gut microbiota might not be a simple reflection of microbes in the surrounding water, but instead result from the species-specific diet, gut morphology, trophic level and phylogeny, as shown in previous studies. Besides, taken into account the notable lower OTU numbers identified in samples S1 and S2 (Table 1), the possibility that these samples are in poor health status (although not visible in this study) is also considered. Nevertheless, these speculations also to some extent implied that the HTS approach from five random individuals employed in this work indeed underestimates the bacterial diversity.

Although the samples detected in this work showed the richness and diversity of gut microbiota, they are relative highly consistent in biological functions. Both KEGG and COG analysis revealed that the most abundant KOs related to membrane transport and metabolism, participate in the amino acid metabolism and transport process in *P. clarkii*. Generally, micro-organisms engaged in a significant part of the animal food chain participating in the material circulation and energy flow in the culture system; on the other hand, played an important role in maintaining ecological balance and optimizing environmental quality (Lee et al. 2014). Studies have indicated that virus infection could facilitate host membrane transport as their genomes and proteins need to be transported across plasma membrane during budding process (Wang et al. 2019). The significant high abundance of gene involved in membrane transport processing observed in this work reflected the role of gut microbial participate in material absorption and energy exchange activity in *P. clarkii*. Unfortunately, whether this positive participation was affected by the CR model farming was remaining unclear. Nevertheless, our ongoing research involved in the comparison analysis of *P. clarkii* gut microbiota among growth conditions will probably enhance our understanding.

Overall, here we have demonstrated that *P. clarkii* within CR model farming in some respects develop a gut microbiota similar to that of crayfish collected recently from their natural habitat, while significant high abundance of specific population (e.g. *Candidatus Bacilloplasma* and *Ornithinibacter*) were also observed. It is unequivocal that the molecular approach such as 16S HTS proved to be a powerful tool for identifying the microbial diversity of intestine in host–microbiota studies.

## Supplementary information

**Additional file 1: File S1.** Summary of OTU numbers and classification at the different levels (phylum, class, order, family and genus).

**Additional file 2: Figure S1.** Rarefaction analysis of V3-V4 Illumina MiSeq sequencing reads of the 16S rRNA gene in different gut ecosystems among the samples. Rarefaction curves at a cutoff level of 3% were constructed at a 97% sequence similarity cutoff value in Mothur. **Figure S2.** Rank-Abundance curves based on OTU level among the samples; **Figure S3**. Hierarchical clustering tree at the OTU level shows the relationship of the gut microbiota of the crayfish farming in CR model. Gut microbiota trees were generated using the UPGMA (unweighted pair group method with arithmetic mean) algorithm based on the Bray-Curtis distances generated by Mothur; **Figure S4**. Phylogenetic tree showing the phylogenetic relationship among the samples in this study on Phylum bar. All bootstrap values > 50% was shown on the tree; **Figure S5**. Cluster of orthologous groups (COG) classification of putative proteins.

**Additional file 3: File S2.** Raw abundance data of the predicted functions. KOs in KEGG level 1 and level 2, as well as level 3 are listed.

## Data Availability

The data supporting our finding included in the manuscript.

## References

[CR1] Abdelrhman KF, Bacci G, Mancusi C, Mengoni A, Serena F, Ugolini A (2016). A first insight into the gut microbiota of the sea turtle *Caretta caretta*. Front Microbiol.

[CR2] Anastacio PM, Frias AF, Marques JC (1999). CRISP (crayfish and rice integrated system of production): 1. modelling rice (*Oryza sativa*) growth and production. Ecol Model.

[CR3] Andersson AF, Lindberg M, Jakobsson H, Backhed F, Nyren P, Engstrand L (2008). Comparative analysis of human gut microbiota by barcoded pyrosequencing. PLoS ONE.

[CR4] Augusto E, Serrano J (2012). Changes in gut evacuation time of larval mud crab, *Scylla serrata* (Crustacea: portunidae) fed artificial plankton or live food. Aquacult Aquar Conserv Legis.

[CR5] Barbee GC, Santer MM, McClain WR (2010). Lack of acute toxicity of an anthraquinone bird repellent to non-target crayfish (*Procambarus clarkii*) associated with rice-crayfish crop rotations. Crop Prot.

[CR6] Becker AA, Hesta M, Hollants J, Janssens GP, Huys G (2014). Phylogenetic analysis of faecal microbiota from captive cheetahs reveals under representation of Bacteroidetes and Bifidobacteriaceae. BMC Microbiol.

[CR7] Burns AR, Stephens WZ, Stagaman K, Wong S, Rawls JF, Guillemin K, Bohannan BJ (2016). Contribution of neutral processes to the assembly of gut microbial communities in the zebrafish over host development. ISME J.

[CR8] Cai C, Li G, Zhu J, Peng L, Li J, Wu Q (2019). Effects of rice-crawfish rotation on sail physicochemical properties in Jianghan Plain. Acta Pedol Sin.

[CR9] Cao CG, Jiang Y, Wang JP, Yuan PL, Chen SW (2017). “Dual character” of rice-crayfish culture and strategy for its sustainable development. Chin Eco-Agri.

[CR10] Carreira BM, Segurado P, Laurila A, Rebelo R (2017). Can heat waves change the trophic role of the world’s most invasive crayfish? diet shifts in *Procambarus clarkii*. PLoS ONE.

[CR11] Chen X, Di P, Wang H, Li B, Pan Y, Yan S, Wang Y (2015). Bacterial community associated with the intestinal tract of Chinese mitten crab (*Eriocheir sinensis*) farmed in Lake Tai China. PLoS ONE.

[CR12] Cresci GA, Bawden E (2015). Gut microbiome: what we do and don’t know. Nutr Clin Pract.

[CR13] Di PP, Chen XB, Sun GW, Xiao JZ, Pan YJ (2014). Constitution analysis of intestinal dominant bacteria community in cultured Chinese mitten crab (*Eriocheir sinensis*). J Microbiol.

[CR14] Dong YY, Chen C, Ren J, Liu ZM (2014). Immunomodulatory effects of lactic acid bacteria on gut-associated immune system: research progress. Chin J Microecol.

[CR15] Du ZQ, Leng XY, Shen XL, Jin YH, Li XC (2017). Identification and characterization of lymph organ microRNAs in red swamp crayfish, *Procambarus clarkii* infected with white spot syndrome virus. Fish Shellfish Immunol.

[CR16] Egerton S, Culloty S, Whooley J, Stanton C, Ross RP (2018). The gut microbiota of marine fish. Front Microbiol.

[CR17] Feng GZ, Zou YX, Wang YL, Wu YT, Shi Y (2019). Screening and identification of xylanase-producing strains isolated from crayfish intestine. Microbiol Chin.

[CR18] Gómez GD, Balcázar JL (2008). A review on the interactions between gut microbiota and innate immunity of fish. FEMS Immunol Med Microbiol.

[CR19] Gu S, Chen D, Zhang JN, Lv X, Wang K, Duan LP, Nie Y, Wu XL (2013). Bacterial community mapping of the mouse gastrointestinal tract. PLoS ONE.

[CR20] Henning SM, Yang J, Shao P, Lee RP, Huang J, Ly A, Hsu M, Lu QY, Thames G, Heber D, Li Z (2017). Health benefit of vegetable/fruit juice-based diet: role of microbiome. Sci Rep.

[CR21] Hoyoux C, Zbinden M, Samadi S, Gaill F, Compère P (2009). Wood-based diet and gut microflora of a galatheid crab associated with pacific deep-sea wood falls. Mar Biol.

[CR22] Kostanjsek R, Strus J, Avgustin G (2007). “*Candidatus* Bacilloplasma” a novel lineage of Mollicutes associated with the hindgut wall of the terrestrial isopod *Porcellio scaber* (Crustacea: isopoda). Appl Environ Microbiol.

[CR23] Lee WJ, Hase K (2014). Gut microbiota-generated metabolites in animal health and disease. Nat Chem Biol.

[CR24] Li Q, Xu L, Xu L, Qian Y, Jiao Y, Bi Y, Zhang T, Zhang W, Liu Y (2018). Influence of consecutive integrated rice-crayfish culture on phosphorus fertility of paddy soils. Land Degrad Dev.

[CR25] Li X, Yu Y, Li C, Yan Q (2018). Comparative study on the gut microbiotas of four economically important Asian carp species. Sci China Life Sci.

[CR26] Liu Z, DeSantis TZ, Andersen GL, Knight R (2008). Accurate taxonomy assignments from 16 s rRNA sequences produced by highly parallel pyrosequencers. Nucleic Acids Res.

[CR27] Meziti A, Ramette A, Mente E, Kormas KA (2010). Temporal shifts of the Norway lobster (*Nephrops norvegicus*) gut bacterial communities. FEMS Microbiol Ecol.

[CR28] Pan H, Li Z, Xie J, Liu D, Wang H, Yu D, Zhang Q, Hu Z, Shi C (2019). Berberine influences blood glucose via modulating the gut microbiome in grass carp. Front Mocrobiol.

[CR29] Quast C, Pruesse E, Yilmaz P, Gerken J, Schweer T, Yarza P, Peplies J, Glöckner FO (2013). The SILVA ribosomal RNA gene database project: improved data processing and web-based tools. Nucleic Acids Res.

[CR30] Roeselers G, Mittge EK, Stephens WZ, Parichy DM, Cavanaugh CM, Guillemin K, Rawis JF (2011). Evidence for a core gut microbiota in the zebrafish. ISME J.

[CR31] Rungrassamee W, Klanchui A, Maibunkaew S, Karoonuthaisiri N (2016). Bacterial dynamics in intestines of the black tiger shrimp and the Pacific white shrimp during *Vibrio harveyi* exposure. J Invertebr Pathol.

[CR32] Shen H, Hu Y, Ma Y, Zhou X, Xu Z, Shui Y, Xu P, Sun X (2014). In-Depth transcriptome analysis of the red swamp crayfish *Procambarus clarkii*. PLoS ONE.

[CR33] Si G, Peng C, Yuan J, Xu X, Zhao S, Xu D, Wu J (2017). Changes in soil microbial community composition and organic carbon fractions in an integrated rice–crayfish farming system in subtropical China. Sci Rep.

[CR34] Si G, Peng C, Xu X, Xu D, Yuan J, Li J (2017). Effect of integrated rice-crayfish farming system on soil physicochemical properties in waterlogged paddy soils. Chin Eco-Agri.

[CR35] Sui L, Ma G, Lu W, Deng Y, Bossier P, Schryver PD (2015). Effect of poly-β-hydroxybutyrate on growth, enzyme activity and intestinal microbial community of Chinese mitten crab, *Eriocheir sinensis* (Milne-Edwards) juveniles. Aquacult Res.

[CR36] Sun CH, Liu HY, Zhang Y, Lu CH (2018). Comparative analysis of the gut microbiota of hornbill and toucan in captivity. MicrobiologyOpen.

[CR37] Terova G, Rimoldi S, Ascione C, Gini E, Ceccotti C, Gasco L (2019). Rainbow trout (*Oncorhynchus mykiss*) gut microbiota is modulated by insect meal from *hermetia illucens* prepupae in the diet. Rev Fish Biol Fisheries.

[CR38] Wang J, Huang Y, Xu K, Zhang X, Sun H, Fan L, Yan M (2019). White spot syndrome virus (WSSV) infection impacts intestinal microbiota composition and function in *Litopenaeus vannamei*. Fish Shellfish Immunol.

[CR39] Wu S, Tian J, Wang G, Li W, Zou H (2012). Characterization of bacterial community in the stomach of yellow catfish (*Pelteobagrus fulvidraco*). World J Microbiol Biotechnol.

[CR40] Wu X, Zhang H, Chen J, Shang S, Wei Q, Yan J, Tu X (2016). Comparison of the fecal microbiota of dholes high-throughput illumina sequencing of the V3–V4 region of the 16S rRNA gene. Appl Microbiol Biotechnol.

[CR41] Yang S, Liebner S, Alawi M, Ebenhoh O, Wagner D (2014). Taxonomic database and cut-off value for processing mcrA gene 454 pyrosequencing data by MOTHUR. J Microbiol Methods.

[CR42] Zeng TL, Ye YF, Mu CK, Wang K, Li RH, Wang CL (2016). Gut microbiota and metabolic phenotype of *Portunus Trituberculatus*. Chin J Anal Chem.

