# Supplementary material for: Gut microbiota of red swamp crayfish Procambarus clarkii in integrated crayfish-rice cultivation model
Source: AMB Express. 2020 Jan 14;10:5. doi: 10.1186/s13568-019-0944-9 (PMC6960274; doi:10.1186/s13568-019-0944-9)
Supplement: Supplementary file 2 — Additional file 2: Figure S1. Rarefaction analysis of V3-V4 Illumina MiSeq sequencing reads of the 16S rRNA gene in different gut ecosystems among the samples. Rarefaction curves at a cutoff level of 3% were constructed at a 97% sequence similarity cutoff value in Mothur. Figure S2. Rank-Abundance curves based on OTU level among the samples; Figure S3. Hierarchical clustering tree at the OTU level shows the relationship of the gut microbiota of the crayfish farming in CR model. Gut microbiota trees were generated using the UPGMA (unweighted pair group method with arithmetic mean) algorithm based on the Bray-Curtis distances generated by Mothur; Figure S4. Phylogenetic tree showing the phylogenetic relationship among the samples in this study on Phylum bar. All bootstrap values > 50% was shown on the tree; Figure S5. Cluster of orthologous groups (COG) classification of putative proteins. [file 13568_2019_944_MOESM2_ESM.doc]

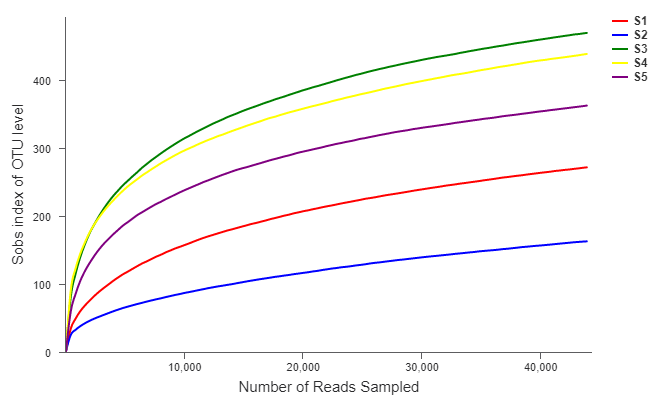


Figure S1 Rarefaction analysis of V3-V4 Illumina MiSeq sequencing reads of the 16S rRNA gene in different gut ecosystems among the samples. Rarefaction curves at a cutoff level of 3% were constructed at a 97% sequence similarity cutoff value in Mothur


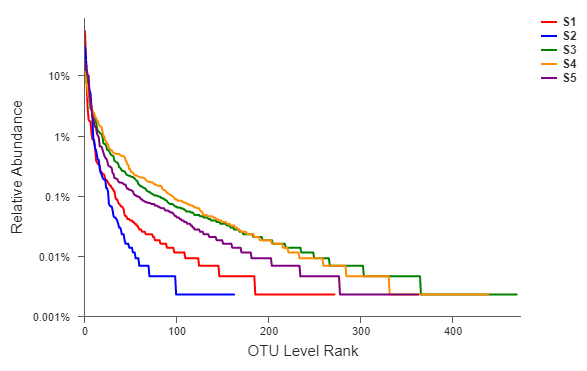


Figure S2 Rank-Abundance curves based on OTU level among the samples


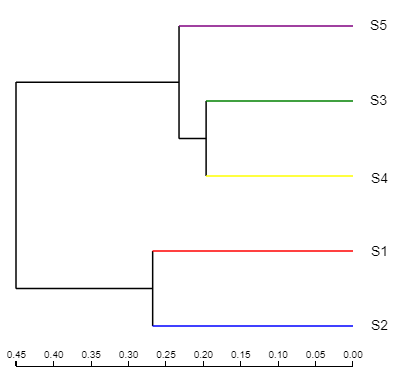


Figure S3 Hierarchical clustering tree at the OTU level shows the relationship of the gut microbiota of the crayfish farming in CR model. Gut microbiota trees were generated using the UPGMA (unweighted pair group method with arithmetic mean) algorithm based on the Bray-Curtis distances generated by Mothur

**
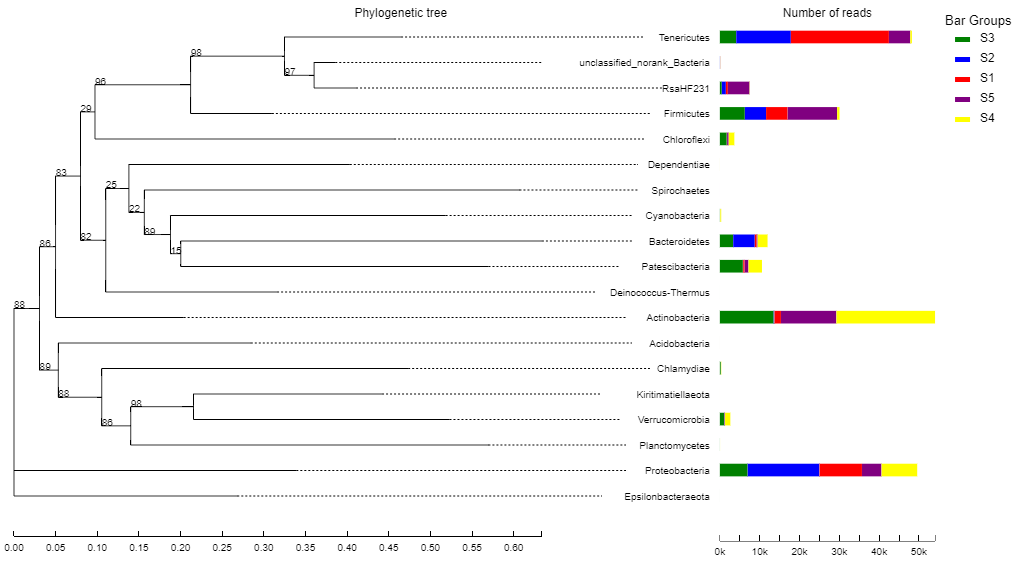
**

Figure S4 Phylogenetic tree showing the phylogenetic relationship among the samples in this study on Phylum bar. All bootstrap values > 50% was shown on the tree

**
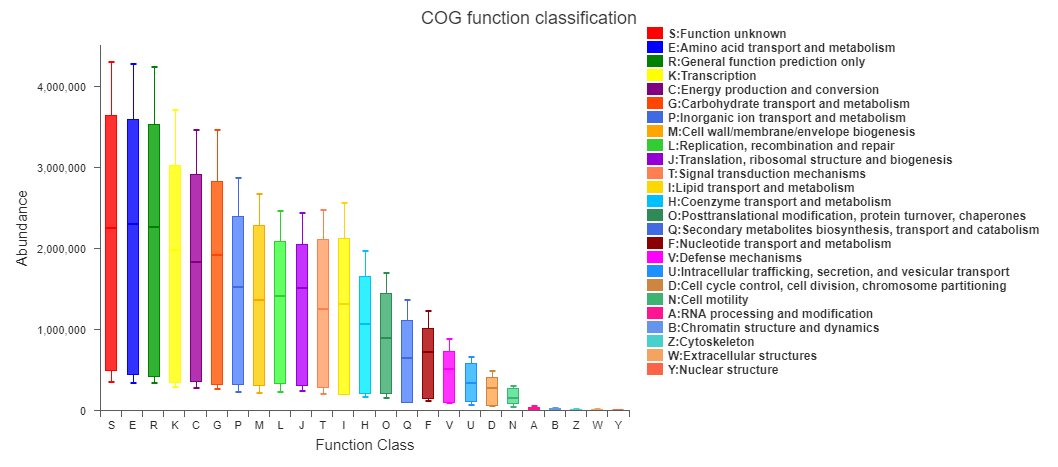
**

Figure S5 Cluster of orthologous groups (COG) classification of putative proteins.
